# Development of Subcutaneous Lecanemab: Establishing the Comparability of Subcutaneous and Intravenous Lecanemab Formulations

**DOI:** 10.1002/alz70859_104694

**Published:** 2025-12-25

**Authors:** Natasha Penner, Sumit Rawal, Jagadeesh Aluri, David Li, Larisa Reyderman, Steven Hersch, Shobha Dhadda, Michael C. Irizarry, Lynn D Kramer

**Affiliations:** ^1^ Eisai Inc., Nutley, NJ USA

## Abstract

**Background:**

A subcutaneous formulation of the anti‐amyloid antibody lecanemab is being developed to improve patient convenience and safety. Herein, we will give an update on the early development history of subcutaneous lecanemab, including establishing the bioavailability and bioequivalence of a lecanemab subcutaneous formulation relative to the intravenous formulation.

**Methods:**

Two single‐dose phase 1 clinical trials in healthy adult participants were performed. The first study was a randomized, parallel‐group study in 60 participants evaluating the absolute bioavailability and pharmacokinetic profile of lecanemab when administered subcutaneously in a 700 mg fixed dose compared to 10 mg/kg of intravenous lecanemab. The second study was a randomized, parallel‐group study in 160 participants to establish the bioequivalence of 720 mg subcutaneous lecanemab when administered by vial compared to autoinjector. Concentrations of lecanemab in serum were measured for 50 days, and noncompartmental PK parameters were calculated. Exposure‐response modeling was conducted to predict efficacy for subcutaneous formulations.

**Results:**

In the bioavailability study (subcutaneous:29; intravenous:30 subjects), mild/moderate injection site reactions were experienced by 6 (20.7%) subjects who received subcutaneous lecanemab, whereas grade 1 and 2 infusion‐related reactions were experienced by 10 (33.3%) subjects who receive intravenous lecanemab. The absolute bioavailability of subcutaneous vial was 49.7% (90% CI: 43.5, 56.8). In the bioequivalence study (vial/syringe:80; autoinjector: 80 subjects), the mean Cmax for vial/syringe (53.7 μg/mL) was lower than the mean Cmax for the autoinjector (67.4 μg/mL). The mean AUC(0‐t) and AUC(0‐inf) were lower for vial/syringe (17,100 and 17,500 ug·h/mL, respectively) compared to the autoinjector (20,600 and 20,900 ug·h/mL, respectively). The half‐life of lecanemab was approximately 7 days in both treatment groups. Autoinjector administration resulted in approximately 25% higher Cmax and 20% higher AUC compared to administration with vial/syringe. The upper 90% CI of the difference in geometric means were outside the standard reference of 125%, therefore bioequivalence of the autoinjector and vial/syringe administrations was not demonstrated. However, based on these results, exposure‐response models predicted similar amyloid plaque lowering and efficacy for intravenous, subcutaneous vial and autoinjector.

**Conclusions:**

Subcutaneous injection of lecanemab using a vial or autoinjector yielded comparable average steady‐state concentrations to intravenous administration of lecanemab in healthy patients.

